# Nanomaterial-mediated multidimensional regulation of the bone microenvironment: a precise therapeutic strategy for bone metabolic imbalance

**DOI:** 10.1080/10717544.2025.2595324

**Published:** 2025-12-05

**Authors:** Chengyan Guo, Chenyu Zhu, Wenjing Li, Jun Zou, Lingli Zhang

**Affiliations:** a School of Exercise and Health, Shanghai University of Sport, Shanghai, China; b School of Sport, Exercise and Health Sciences, Loughborough University, Loughborough, UK; c College of Athletic Performance, Shanghai University of Sport, Shanghai, China

**Keywords:** Nanomaterials, bone microenvironment, bone metabolism, immunometabolic reprogramming, vascular-osteogenic coupling, lipid metabolism

## Abstract

With the advancement of global population aging, the incidence of skeletal diseases (e.g. osteoporosis, fractures, and osteoarthritis) in clinical diagnosis increases and poses a serious threat to human health. Skeletal diseases usually occur as a result of disturbed cellular metabolism in a specific period or environment. The bone microenvironment, as an important physiological environment of bone tissue, consists of various cell types, and cell–cell interactions play a decisive role in the biological behavior and metabolic regulation of bone cells. Disorders of the bone microenvironment can exacerbate bone diseases. Conventional therapeutic for skeletal diseases often suffer from poor efficacy, low targeting, and side effects. Therefore, a new therapeutic strategy should be developed urgently to improve the existing deficiencies. With the continuous advancement of nanomedicine, the application of nanomaterials provides new research perspectives and application value for the treatment of skeletal diseases. With their unique physicochemical properties, nanomaterials can directly or indirectly mediate the bone microenvironment to regulate the bone metabolic process through self-regulation, drug carriers, and in vivo scaffolds. All the above strategies are extensively explored in this study. In this paper, we systematically summarize the nanomaterials currently used in the clinical treatment of bone diseases and discuss the application strategies of nanomaterials to regulate the bone microenvironment and thus bone metabolism. Moreover, we evaluated the challenges faced by nanomaterials in the clinical treatment of bone diseases. We aim to provide basic theories and new perspectives for the design and development of novel nanomaterials for improved clinical applications.

## 1. Introduction

The human skeleton is a multifunctional organ composed of multiple cell types that work synergistically to maintain skeletal and systemic mineral homeostasis and perform critical mechanical and endocrine functions (Fornetti et al. 2018). Bone metabolic homeostasis is critical for the formation of bone morphology and structure, as well as the repair of bone tissue. The bone microenvironment, as a complex biological environment, precisely regulates bone metabolism through multicellular synergies and molecular networks (Heaney 2007). Specifically, osteoblast (OB)-mediated bone formation function and osteoclast (OC)-mediated bone resorption function are critical for maintaining the balance of bone metabolism (Hawkes and Mostoufi-Moab 2019). In addition, the coupled activities of other elements in the bone microenvironment (e.g. OBs, mesenchymal stem cells, immune cells, and neural cells) play an essential role in balancing the bone metabolic process (Bertels et al. 2024). Disturbances in any of these elements of the bone microenvironment may lead to imbalances in bone metabolism, which in turn may lead to the development of skeletal diseases, such as osteoporosis (OP), osteoarthritis, osteosclerosis, and bone tumors (Wu et al. 2024).

Currently, the most common treatment modalities for bone diseases are medication, surgery, and physical therapy (Mura et al. 2013). Physiotherapy is an important therapeutic strategy in modern traditional medicine that uses noninvasive, nonpharmacological treatments to relieve pain and assist in the treatment of pain caused by bone disorders through physical factors such as sound, light, cold, heat, electricity, and force (Mao et al. 2021). In recent years, exercise has been recognized as an important intervention for the treatment and prevention of a wide range of diseases and has been shown to alleviate a wide range of bone disorders. However, the relationship between the exercise load and therapeutic effect without inducing disease progression has not been fully elucidated, and inappropriate exercise prescriptions may instead increase the risk of fracture (Watson et al. 2015). Surgical treatment, as an important intervention for skeletal diseases, is applicable mainly to severe skeletal lesions caused by specific pathological factors (Voutzoulias et al. 1997; Landa et al. 2007; Vidal et al. 2020; Li et al. 2022), but it still inevitably causes different degrees of medically induced injuries to local tissues (Liu et al. 2017). At present, pharmacotherapy, including topical, oral, and injectable interventions, remains the preferred strategy for the clinical management of skeletal disorders. Although oral medications have the advantage of therapeutic convenience, their low bioavailability predisposes patients to poor therapeutic outcomes (Miladi et al. 2013). In addition, exogenous injectable drugs are easily recognized and cleared by the host immune system, triggering a strong immune rejection reaction, which leads to a significant reduction in the efficiency of targeted delivery to bone tissue(Hirabayashi et al. 2001; Murphy et al. 2007; Low and Kopeček 2012). Considering these limitations, the development of novel therapeutic strategies with bone-targeting properties that can effectively regulate bone metabolic pathways and promote bone tissue regeneration has become an urgent need for current research (Dvir et al. 2011).

In recent years, the emergence of nanotechnology has led to various nanomaterials that have been widely used in the diagnosis, treatment, and regeneration of living organisms (Ye et al. 2020). Its advantages include enhanced drug-carrying capacity (Ma et al. 2015; Lee et al. 2016), prolonged drug residence time, efficient release kinetics, (Gan et al. 2013) precise targeting ability, and decreased side effects (Mura et al. 2013). Therefore, based on the interaction mechanism between the bone microenvironment and bone metabolism, designing and constructing nanosystems that can modulate the microenvironment of bone tissues is a therapeutic strategy for bone diseases with potential clinical applications. However, systematic reviews on the role of nanotechnology in the bone microenvironment-mediated regulation of bone metabolism are lacking. Therefore, in this work, we systematically summarize the nanomaterials that are currently used in the clinical treatment of skeletal diseases and explore the application strategies of nanomaterials to regulate the bone microenvironment and thus bone metabolism. Moreover, we assess the challenges faced by nanomaterials in the clinical treatment of skeletal diseases. This work provides not only a theoretical basis for the development of new materials and delivery systems for the treatment of bone-related diseases but also new ideas for cutting-edge research in the field of bone tissue repair and regeneration from a multidisciplinary perspective.

## 2. Types of bone-related nanomaterials

**Figure 1.**
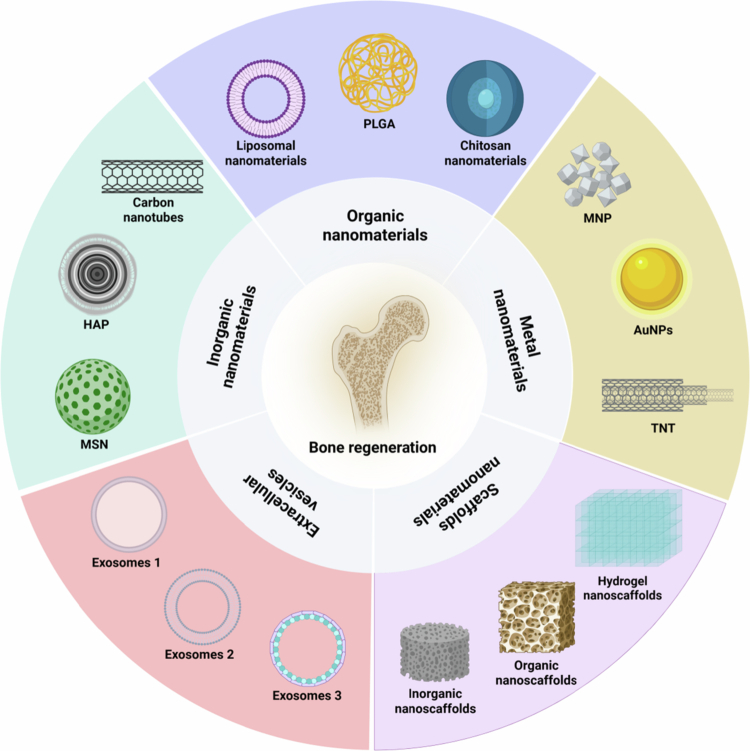
Classification of nanomaterials in bone disease treatment and tissue engineering. Organic nanomaterials include liposome nanoparticles, poly(lactic-co-glycolic acid) (PLGA) nanoparticles, and chitosan nanoparticles. Inorganic nanomaterials include mesoporous silica nanomaterials (MSN), nanohydroxyapatite (nHAP), and carbon nanotubes. Metallic nanomaterials include gold, silver, magnetic oxide particles, and titanium nanotubes. In addition, extracellular vesicles and nanoscaffolds have become advanced nanomaterials with a wide range of applications in bone tissue engineering. All of these nanomaterials can be used as direct or indirect carriers to regulate bone metabolism and promote bone regeneration through drug delivery, structural support, or bioactive signalling. (This image was created and painted from BioRender.).

Nanomaterials are biological materials with dimensions between 1 and 100 nm, which are considered the most central part of the field of nanomedicine owing to their unique physicochemical properties (Ali et al. 2021). At this submicron scale, the properties of the material change significantly because of quantum effects and increased surface-to-volume ratios (Cheng et al. 2021). These unique properties make nanomaterials particularly promising for applications in the treatment of skeletal diseases and bone tissue engineering. Nanomaterials cannot only deliver some biologically active molecules (e.g. drugs, genes, and growth factors) to the target region with precision and controlled release but also reduce their exposure and biological degradation to nontargeted cells (Valcourt et al. 2020). We focus on the types of nanomaterials currently used for the treatment of bone metabolic diseases, including organic nanomaterials, inorganic nanomaterials, metal nanomaterials, extracellular vesicles (EVs), and nanoscaffolds (Figure 1).

### 2.1 Organic nanomaterials

Organic nanomaterials are generally nanoscale materials formed from organic compounds through assembly and processing, specifically including liposomal nanoparticles (NPs), poly(lactic-co-glycolic acid) (PLGA) NPs, and chitosan (CS) NPs. Among them, liposomal NPs are the most widely used nanomaterials in current clinical practice (Thi et al. 2021). Liposomes not only exhibit a high degree of simplicity in processing and synthesis but also possess hydrophilic and lipophilic characteristics, which together give liposomes excellent biocompatibility and degradability. Owing to these properties, liposomal nanomaterials are commonly used in a wide range of applications, such as drug delivery, bioimaging, and phototherapeutic medicine (Battaglia and Gallarate 2012). For applications in the field of bone tissue, liposomal nanomaterials are convenient, safe, and economical biomaterials for inducing endogenous bone tissue growth and bone regeneration. Teng et al. constructed a nanoliposome system for baicalein (BCL) delivery using a reverse distillation technique (Minhua et al. 2022). By in vitro cell realization, they showed that this delivery system significantly promoted OB differentiation and maturation owing to the enhanced encapsulation and electrostatic stability of liposomes.

Among various types of polymeric nanomaterials, PLGA is a hydrophobic polymer material with good biocompatibility and high degradability (Dayanandan et al. 2023). The material can be coupled with specific ligands to achieve targeted functionalization modifications (Betancourt et al. 2009). PLGAs also exhibit excellent release kinetics, enabling the precise delivery of drugs and bioactive factors to the target region, as well as long retention and controlled release (Vert et al. 1994). These properties can significantly reduce the amount of drug and biokinetic factors and greatly improve the bioavailability of PLGA (Danhier et al. 2012). In addition, the byproducts generated during the degradation of PLGA can be effectively eliminated through in vivo metabolic pathways, thus reducing the toxicity to cells and enhancing its biosafety (Acharya and Sahoo 2011). In the field of bone medicine, in addition to the effective delivery of antiresorptive drugs (e.g. bisphosphonates), PLGA NPs can deliver bone-related hormones and proteins, such as parathyroid hormone (PTH) and bone morphogenetic protein (BMP)-2, which can effectively protect biologically active molecules from degradation by enzymes and can significantly increase the biosafety of OBs (Danhier et al. 2012; Yilgor et al. 2009).

CS is a naturally occurring polysaccharide derived mainly from chitin in crustaceans, insects, and fungi (Saini et al. 2015). CS-NPs are widely used for the delivery of various drugs and other biomolecules given their small size, high encapsulation efficiency, and high loading capacity (Zhao et al. 2018). Moreover, CS-NPs can be used as effective carriers for gene delivery. CS has positively charged amino groups in its molecular backbone, which can be stably bound to negatively charged nucleotides by electrostatic interactions, thus effectively protecting the nucleotides from degradation by the corresponding enzymes (Mao et al. 2010). However, the cell internalization efficiency of CS-NPs is inadequate, and this limitation directly affects their application in gene delivery. To solve the above problem, Zhao et al. interacted CS-NPs with polyethyleneimine and formed a new complex, which was subsequently applied to a rat model to deliver the BMP-2 gene (Zhao et al. 2016). The results showed that the complex could significantly promote the internalization efficiency of cells and promote bone formation in rats in vivo. Thus, this approach not only improves the stability and transfection efficiency of the gene delivery system but also provides an efficient nanodelivery platform for the modern gene therapy bone regeneration field.

### 2.2 Inorganic nanomaterials

Inorganic nanomaterials are composed mainly of inorganic substances, including mesoporous silica NPs (MSNs), nanohydroxyapatite (nHAP), and carbon nanotubes. They have attracted much attention in the field of bone tissue repair and regeneration owing to their high structural similarity to the natural mineral composition of bone tissue and their good mechanical properties. During the preparation of inorganic nanomaterials, physical parameters, such as pore structure, pore size, and specific surface area, can be regulated to achieve efficient encapsulation and delivery of loaded substances (genes, proteins, and small-molecule drugs).

MSNs are a class of porous nanomaterials containing numerous mesopores with a diameter ranging from 2 to 50 nm (Feng et al. 2023). The highly ordered mesoporous structure of these nanomaterials allows them to carry various drugs and therapeutic agents on their own, which can be utilized to accomplish bone repair and regeneration by promoting OB-mediated bone formation or inhibiting OC-induced bone resorption (Larsson and Fazzalari 2014; Xu et al. 2023). In addition, the silanol groups on the surface of MSN not only endow the material with good biocompatibility to interact with body fluids and internalize into natural bone tissues in the body but also can combine with different groups that have multiple roles in recruiting or binding osteogenic peptides and growth factors in vivo and promoting bone regeneration (Chen et al. 2019). Wu et al. used well-established sol–gel and supramolecular surfactants to promote bone regeneration through the combination of MSN with OB-mediated bone formation or the inhibition of OC-induced bone resorption using chemical techniques (Wu and Chang 2012). Chemical techniques, such as the use of MSNs and calcium ions, were adopted to successfully construct mesoporous bioactive glass nanospheres by combining MSNs with calcium ions. The nanospheres could accomplish the efficient delivery of drugs and growth factors and significantly enhance the proliferation and differentiation of OB.

Hydroxyapatite (HAP) is a naturally occurring inorganic material with a crystal structure, size, morphology, and chemical composition similar to those of natural bone minerals (Bharadwaz and Jayasuriya 2020). They exhibit a strong affinity for type I collagen, which can effectively promote the integration and regeneration of bone tissue (Lara-Ochoa et al. 2021). In clinical applications, nHAP is usually used as a bone repair material for the treatment of OP, bone defects, and other related diseases (Swetha et al. 2010). nHAP can be constructed into composites with novel functional properties by compositing with other biopolymers to effectively meet the requirements of bone regeneration. These biocomposites exhibit efficient mechanical properties and osteoconductivity and remain stable for a long time in acidic or alkaline environments in vivo, preventing the biodegradation process in vivo (Sun et al. 2011).

### 2.3 Metal nanomaterials

With the rapid development and deep integration of the fields of nanomedicine and materials science, metallic nanomaterials have become a hot research topic of great interest. Many metallic nanomaterials, including Au, Ag, Mg, Ti, Ni, and magnetic NPs (MNPs), have been reported in depth by scholars. AuNPs are colloidal particles composed of gold atoms with diameters ranging from a few nanometers to hundreds of nanometers. Owing to their unique physicochemical properties, AuNPs are easy to be engineered into various sizes and shapes and can be multifunctionalized by surface modification of functional groups to adapt to different application scenarios (Ghosh et al. 2008). AuNPs can effectively regulate the activities of OB and OC and reduce the differentiation of bone marrow mesenchymal stem cells (BMSCs) into lipid-forming cells (Sul et al. 2010). However, large-sized AuNPs are difficult to internalize by cells, tend to accumulate in the liver and spleen, and exhibit certain cytotoxicity (Chithrani et al. 2006). In addition, an excessively high concentration of AuNPs may lead to apoptosis, whereas an extremely low concentration has no significant effect on osteogenic differentiation (Jiang et al. 2023). Therefore, optimizing the size, shape, and concentration of AuNPs has become a key factor in developing AuNPs. Currently, researchers are still exploring the optimal application strategy for AuNPs in the treatment of bone-related diseases, which are expected to become highly promising functional materials for biomedical applications in the future.

In recent years, extensive research has focused on synthesizing AgNPs with controllable size and shape for applications such as bone tissue repair and wound healing, enhancing vaccine immunogenicity, and diabetes treatment (Asgary et al. 2016; Saratale et al. 2017; Shanmuganathan et al. 2019). Additionally, AgNPs have garnered significant attention for their broad-spectrum and highly effective antibacterial and antitumor activities (Otari et al. 2015). In terms of antimicrobial effects, AgNPs demonstrate significant inhibitory activity against diverse pathogens, including bacteria, fungi, and viruses. This may be attributed to multiple antimicrobial mechanisms, primarily involving disruption of the cell wall and cell membrane structures, induction of elevated intracellular reactive oxygen species (ROS) levels, and damage to DNA structures (Xu et al. 2020). Nandi et al. employed electrolytic deposition to form a AgNP coating on stainless steel implants (Nandi et al. 2018). In a rabbit osteomyelitis model, this coating significantly enhanced osteoblast activity and angiogenesis due to the antibacterial and anti-inflammatory properties of low-dose AgNPs. Ultimately, effective healing of the infected bone defect was achieved by day 42. In antitumor applications, the biological effects of AgNPs strongly depend on their physicochemical parameters (e.g. particle size, morphology, and surface charge), enabling them to specifically target and kill various types of cancer cells (El Badawy et al. 2011; Dziedzic et al. 2016). The intrinsic mechanism involves inducing tumor cell apoptosis and necrosis by disrupting the cancer cell ultrastructure, inducing reactive oxygen species (ROS) production and DNA damage, and inhibiting key enzyme activities and signaling pathways (Eom and Choi 2010; Bethu et al. 2018). Additionally, AgNPs can block tumor cell survival and invasion by suppressing angiogenesis (Homayouni-Tabrizi et al. 2019). For example, Kovács et al. found that citrate-coated AgNPs were effectively internalized by osteosarcoma cells (Kovács et al. 2016). The core mechanism involved inducing mitochondrial stress, manifested as a decrease in the mitochondrial membrane potential, ultimately leading to cancer cell apoptosis.

Magnesium (Mg) serves as the primary storage element in the skeletal system, playing a crucial role in maintaining bone structure and metabolic homeostasis. Research indicates that Mg²⁺ participates in various cellular biological processes, exhibiting significant physiological importance in bone tissue repair and regeneration by reducing inflammatory responses, stimulating osteoblast differentiation, and enhancing angiogenesis capabilities (Banai et al. 1990; Liang et al. 2020). However, the highly reactive chemical nature of metallic magnesium causes it to spontaneously oxidize in air, forming a magnesium oxide surface layer. This poses significant challenges for the synthesis, processing, and storage of MgNPs (Czerwinski 2012; Dai et al. 2024). With advances in materials science, the development of magnesium-derived biomaterials – including nanoalloys, nanocomposites, nanometallic-organic frameworks (MOFs), and nanocomposites – has emerged as a key research direction in bone tissue engineering (Zhou et al. 2024). These materials combine suitable mechanical properties with excellent biocompatibility, offering broad prospects for treating bone-related diseases. For example, magnesium-metal-organic frameworks (Mg-MOFs) constructed via physicochemical crosslinking can induce macrophage polarization toward the M2 phenotype, thereby promoting early osteogenic differentiation. In rat femoral defect models, these frameworks demonstrate significant bone regeneration capabilities (Li et al. 2023).

Ti metal is known for its high mechanical strength, biocompatibility, and corrosion resistance and is commonly used as a bone implant material, such as in screws, titanium plates, and bone nails (Tschernitschek et al. 2005). TNT is a new type of metallic nanomaterial prepared by nanotechnology, and the process of its preparation enables structural delamination and a significant increase in surface roughness, which enhances the interaction with surface-adherent cells and promotes the deep fusion of TNT with bone tissue (Mendonça et al. 2008). Moreover, TNT exhibits excellent adhesion to OB, which can promote the proliferation and differentiation of OB cells and improve the repair and regeneration efficiency of bone tissue (Gulati et al. 2012). TNT nanomaterials are not only an advantageous contender as bone implant materials but are also highly regarded in the field of drug delivery. In conclusion, this material has great application value in the field of bone tissue engineering.

### 2.4 EVs

EVs are nanometer-sized, double-layered lipid‒membrane vesicles released from most types of cells and include mainly exosomes, microvesicles, and apoptotic vesicles (Nowak et al. 2023). The exterior of these vesicles is enriched with transmembrane proteins, conferring them the ability to participate in intercellular communication (Liu et al. 2023). In the field of drug delivery, researchers are working on developing drug delivery strategies based on EVs (Herrmann et al. 2021). The advantage of this strategy is that EVs, as natural biovectors, do not trigger immune rejection during delivery (Cui et al. 2022). In the bone remodeling microenvironment, EVs can deliver specific proteins (e.g. tendon protein C and semaphorin 4D) and other growth factors (e.g. BMPs 1–7 and transforming growth factor β1) to OB to regulate bone formation. In addition, EVs can deliver cytokines, such as receptor activator of nuclear factor kappa-B (RANK) and RANK ligand (RANKL), as well as microRNAs, such as miR-218 and miR-148a, to promote OC differentiation and bone resorption function (Liu et al. 2018). In recent years, research on EV-based regulation of bone homeostasis and regeneration has become a cutting-edge direction in the field of bone tissue, and it may have broader application prospects in this field in the future (Fang et al. 2024).

### 2.5 Nanoscaffolds

Bone regeneration strategies with nanoscaffolds are also considered important in bone tissue engineering, given that they have successfully improved the limitations and drawbacks of allogeneic and homograft bone grafting methods and provided stable environmental conditions for bone defect healing (Cui et al. 2019). Various scaffold fabrication techniques, including spin-coating, template methods, electrostatic spinning, UV irradiation, lithography, compression, filtration, and emerging 3D printing technologies, have been widely used to fabricate bone regenerative scaffolds with adhesive and mechanical properties (Deng et al. 2019). The application strategy of nanoscaffolds is to load cells and growth factors into the pore structure of the nanoscaffolds by integrating and implanting them into the bone defect site to stimulate the proliferation and differentiation of osteogenic bone (Jeon et al. 2012). However, the selection of nanoscaffold materials is a critical factor in achieving bone remodeling and regeneration. Ideal bioscaffolds can exhibit good biocompatibility and osteoconductivity with host tissues and maintain the activity of cells and biokines, thus accelerating bone regeneration (Koushik et al. 2023). Significant progress has been made in the research of nanoscaffolds in the field of bone regeneration, but some challenges remain in their clinical translational applications. In the future, researchers should pay considerable attention to evaluating the effects of various scaffold materials on in vivo toxicity and studying vascular nerve regeneration mechanisms to provide a theoretical basis and technical guidance for the clinical application of bioactive nanoscaffold composites (Marrella et al. 2018).

## 3. Mechanisms of nanomaterials regulating bone metabolism through multitargets in the bone microenvironment

### 3.1 Introduction to the skeletal microenvironment

**Figure 2.**
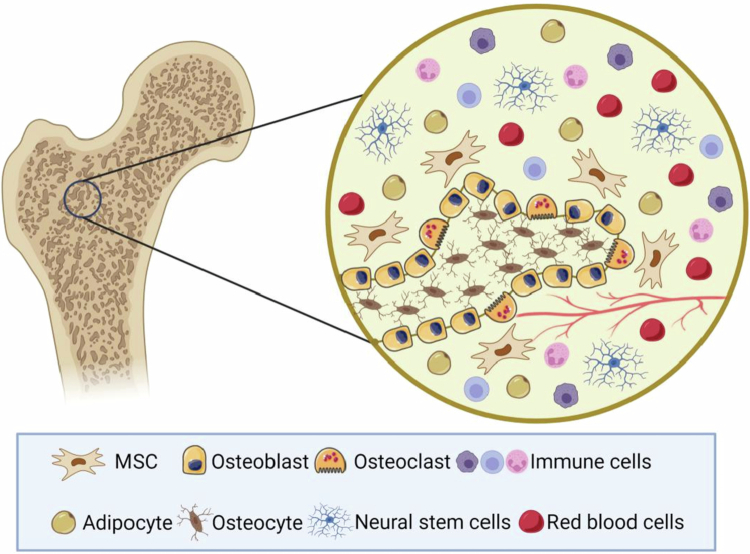
Cellular and structural components of the bone microenvironment. The bone microenvironment is composed of a highly regulated network of different cell types and tissues, including mesenchymal stem cells, osteoblasts, osteoclasts, osteocytes, immune cells, adipocytes, neuronal cells, and the bone vascular system. These cells interact in a highly balanced and tightly regulated manner by secreting a variety of growth factors and signaling molecules to maintain bone metabolic homeostasis and coordinate the bone remodeling process. (This image was created and painted from BioRender.

Bone is a highly mineralized specialized connective tissue with a complex multilayered structure (Reznikov et al. 2014). As a metabolically active tissue, it not only provides stable support and motility for the body but also maintains a dynamic balance of minerals (Meng et al. 2024). In this dynamic process, various cells in the bone and the growth factors they secrete play crucial roles, which together constitute the skeletal microenvironment (Salhotra et al. 2020). BMSCs, as pluripotent stem cells, have the potential to differentiate into osteogenic or adipocyte lineages (Chai 2022). A dynamic equilibrium exists between the osteogenic and adipogenic differentiation of BMSCs, which is influenced by strict molecular regulatory mechanisms (Lin et al. 2019). OB, as a direct participant in bone metabolic homeostasis, is mainly responsible for synthesizing new bone matrix and regulating bone mineralization processes (Rutkovskiy et al. 2016). Bone marrow adipocytes (BMAs) are among the most abundant cell types in the bone marrow and can release adipokines through paracrine effects and activate the peroxisome proliferator-activated receptor *γ* (PPARγ) signaling pathway to promote adipogenesis. However, the expansion of bone marrow adipose tissue (BMAT) tends to affect the differentiation and function of OB and other cells, which can increase the risk of bone loss. OC is the only bone resorptive cell in the bone microenvironment, and it dissolves the bone matrix mainly by acidifying the microenvironment. In the presence of colony-stimulating factor (CSF)-1, bone marrow-derived macrophages (BMMs) and hematopoietic stem cells (HSCs) proliferate and differentiate into OC precursor cells (OPCs), which further differentiate and fuse with RANKL. Eventually, mature multinucleated OCs are formed to perform bone resorption functions (Song et al. 2019; Søe et al. 2021). HSCs in the bone marrow can differentiate into immunocompetent subpopulations of leukocytes and erythroid hematopoietic cells in specific environments (Mendoza-Reinoso et al. 2020), including macrophages, T cells, and B cells. Immune cells can participate in the maintenance of bone microenvironmental homeostasis through various secreted cytokines (e.g. RANK L, TNF-*α*, and IL-10) that form a complex regulatory network with OBs, OCs, and vascular endothelial cells (ECs). Extracellular matrix (ECM) is also an important component of the bone microenvironment. It not only provides strong external support and protection for cell survival but also serves as a signaling hub for the dynamics of intercellular interactions, regulating cell fate and bone tissue function (Lu et al. 2018; Baslé et al. 1998). Meanwhile, the highly developed vascular system in the bone microenvironment not only provides nutritional support for the normal physiological activities of bone tissue but also regulates bone formation through vascular–osteogenic coupling mechanisms (Rather et al. 2019). Nerve fibers in bone tissue, including sensory, sympathetic, and glutamatergic nerves, are involved in bone metabolism by secreting and releasing neurotransmitters, neuropeptides, and neurotrophic factors (Elefteriou 2018). Therefore, the complex bone microenvironment plays a decisive role in maintaining the balance of bone metabolism. Multitarget regulation of the bone microenvironment based on nanotechnology is expected to provide a new research direction for the treatment of skeletal diseases by directly or indirectly affecting the activity and differentiation process of OB and OC (Figure 2).

### 3.2 Nanodelivery systems regulate osteogenic differentiation: from osteogenic signaling pathway activation to biomineralization guidance

OB is mainly responsible for producing ECM proteins, regulating matrix mineralization, controlling bone remodeling, influencing OC differentiation, and playing an important role in bone homeostasis (Corrado et al. 2017). One of the key factors in achieving bone regeneration is improving the osteogenic differentiation of BMSCs. However, BMSCs are regulated directly or indirectly by multiple signaling pathways, and targeting osteogenesis-related pathways to promote osteogenic differentiation is the key to treating OP. Nanomaterials are particularly important in delivering various bioactive molecules to promote osteogenic differentiation owing to their good stability, high biocompatibility, high drug-carrying capacity, and excellent slow release function.

Icariin (ICA) is a drug belonging to class IV biopharmacological classification system and is commonly used clinically for the treatment of bone metabolism-related diseases (Zhang et al. 2014). However, the drug suffers from the disadvantages of low solubility, poor permeability, and low bioavailability, which severely limit its clinical efficacy. To improve the therapeutic efficacy of ICA, Dong et al. successfully infiltrated ICA into fetal bovine serum exosomes by coincubation, thus synthesizing a FBS EXO-ICA nanodelivery system (Dong et al. 2021). Compared to Icariin alone, FBS EXO-ICA effectively increased the expression of the osteogenic markers BMP-2, Runx2, and OPN, promoting OB proliferation and differentiation, while also demonstrating good bioavailability and safety. Li et al. discovered a bioactive glass NP (BGN), which interacted with rat BMSCs by indirectly releasing reactive ions or completing the interaction with rat BMSCs through direct internalization and induced Runx2 and Osterix (Osx) activation of transcription factors to promote the osteogenic differentiation of BMSCs (Meng et al. 2024). These genes subsequently promoted the osteogenic differentiation of BMSCs to a great extent after increasing SiO_2_–CaO synthesis in the BGN, and the upregulated expression of Runx2 and Osx was positively correlated with the CaO content in the BGN. Curcumin (Cur), a polyphenol derived from turmeric plants, has been shown to modulate immune responses, inhibit lipogenic differentiation, and reduce oxidative stress to promote OB differentiation(Dai et al. 2017; Chen et al. 2016). Yang et al. successfully coupled Cur to polyamide resin (PAD) by HCCP bonding and prepared a new type of pH-responsive Cur-loaded nanospheres (HCCP-Cur-PAD, HCP NPs), which was verified for its cytotoxicity (Yang et al. 2023). They found that HCP NPs were safe for application to MC3T3-E1 cells and had no toxic effect on MC3T3-E1 cell viability. In terms of osteogenic differentiation effects, the HCP NP group increased the expression of Runx2 and Osx transcription factors, ALP activity, and calcium deposit formation, and the level of the structural protein COL1A1 in the bone ECM was also significantly elevated compared with that in the free control group. HCP NPs exhibit significant promotion of osteogenic differentiation and bone formation and may become a promising nanodelivery system in the future.

The Wnt/β-catenin signaling pathway plays an important role in promoting bone growth, development, and remodeling (Baron and Kneissel 2013). Sclerostin (SOST), a glycoprotein secreted by mature osteocytes within the bone matrix, inhibits bone formation via the Wnt/β-catenin signaling pathway (Duan and Bonewald 2016; Wang et al. 2014; Pietrzyk et al. 2017). Research indicates that reactivating the Wnt/β-catenin signaling pathway using siRNA adenovirus vectors carrying a silenced SOST gene significantly enhances the expression of the key osteogenic differentiation markers Alp and Runx2, thereby reversing the bone loss induced by Ti particles (Pietrzyk et al. 2017). However, siRNAs have a short half-life and weak transmembrane capacity (Kim et al. 2016; Wang et al. 2012). Therefore, an efficient carrier for delivery and release must be developed urgently. Mora-Raimundo et al. chose MSNs as a nanocarrier for siRNA and coated it with polyetherimide to form a targeting system, which could encapsulate two therapeutic agents, namely, SOST siRNA and osteostatin, through their high loading capacity, further enhancing the synergistic therapeutic effect of bone regeneration (Mora-Raimundo et al. 2019). In vitro and in vivo experiments demonstrated that MSN as carriers of the SOST gene had a more pronounced knockdown effect than viral vectors, and the expression of ALP, a marker of early osteogenic differentiation, increased significantly, which further highlights the positive role of MSN in bone regeneration. Choi et al. investigated the effects of chitosan-conjugated gold nanoparticles AuNPs on the differentiation of human adipose-derived mesenchymal stem cells (hADMSCs). They found that hADMSCs actively internalized chitosan-conjugated AuNPs, triggering intracellular mechanical stress responses. Further mechanistic studies revealed that this process activates the Wnt/β-catenin signaling pathway, significantly upregulating the expression of non-phosphorylated *β*-catenin and promoting its transport from the cytoplasm to the nucleus. This ultimately leads to the stable accumulation of nuclear *β*-catenin. This signaling cascade directly results in a significant increase in the expression of osteoblastic differentiation-related markers ALP, BSP, and OCN, ultimately enhancing the mineralization level of OBs. These findings confirm that chitosan-conjugated AuNPs enhance osteogenic differentiation of hADMSCs and redirect their fate away from adipogenic differentiation (Choi et al. 2015).

The BMP-Smad signaling pathway is a classical pathway that regulates osteogenic differentiation and plays a key role in osteogenic differentiation of stem cells, regeneration of bone tissue, and development of bone-related diseases (Zhang et al. 2023). BMP-2 is a key growth factor that induces osteogenic differentiation and regeneration (Yasko et al. 1992). Dexamethasone (DEX) is a synthetic glucocorticoid and a good inducer in the bone marrow stroma (Kim et al. 2012). Zhou et al. succeeded in constructing a nanodelivery system for the regulation of cellular osteogenic differentiation by coupling a BMP-2 peptide to the surface of a DEX-loaded MSN (DEX@MSNs-pep) (Zhou et al. 2015). DEX@MSNs-pep exhibited excellent dispersion and good biocompatibility. Compared with the BMP-2 peptide or DEX alone, the nanosystem significantly increased the osteogenic differentiation efficiency of the BMSCs and promoted the expression of the osteogenic transcription factor Runx2 and the late osteogenic differentiation-specific marker OCN. Further studies revealed that the BMP-2 peptide specifically recognizes BMP receptors on the cell membrane surface, thereby activating the intracellular BMP-Smad signaling pathway. This process promotes the phosphorylation of Smad1/5/8, which then forms a transcription complex with Smad4 and translocates into the nucleus. This complex exhibits synergistic effects with the dexamethasone-activated glucocorticoid receptor within the nucleus, effectively enhancing osteoblast OB differentiation and in vitro mineralization capacity. Magnetic iron oxide NPs (IONPs) are biomaterials capable of promoting bone regeneration through mechanical stimulation (Henstock et al. 2014). Magnetic field-induced IONPs can promote the differentiation of mouse primary cell BMSCs into OBs (Sun et al. 2014). To further investigate the mechanism of IONPs in bone repair, Wang et al. verified the positive effect of IONPs on the osteogenic differentiation of MSCs by in vitro experiments. Based on genomic sequencing, the long-chain noncoding RNA INZEB2 may play a key role in regulating osteogenic differentiation (Wang et al. 2017). ZEB2, a member of the ZEB protein family, can interact with the BMP receptor protein Smad, which in turn inhibits specific transcriptional processes (Postigo 2003). IONP-treated MSCs upregulate the expression of the long non-coding RNA INZEB2 while downregulating the protein expression of ZEB2, indicating that INZEB2 maintains osteogenic differentiation by negatively regulating ZEB2 expression. Further mechanistic studies revealed that osteogenic differentiation of BMSCs depends on its inhibitory effect on ZEB2; downregulation of ZEB2 effectively releases ZEB2 binding to the phosphorylated Smad protein complex and its transcriptional repression function. This action restores the nuclear localization capacity of the Smad complex, thereby activating the expression of RUNX2, a key transcription factor for osteogenic differentiation, and promoting the directed differentiation of BMSCs toward the OB lineage. These findings reveal the pivotal role of the INZEB2/ZEB2 axis in regulating the BMP-Smads signaling pathway and osteogenic differentiation.

**Figure 3.**
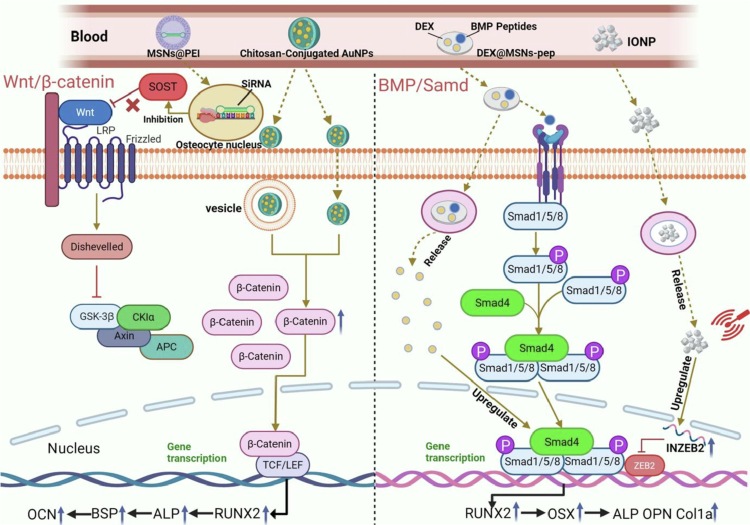
Application of nanomaterials for inducing osteogenic signaling pathways**.** The MSN@PEI nanodelivery system and AuNPs promote osteoblast differentiation and mineralized calcium deposition by activating the Wnt/β-catenin signaling pathway. The DEX@MSN-pep nanodelivery system and IONP enhance the expression of osteogenic differentiation-related genes and promote bone formation by activating the BMP-Smad signaling pathway. (This image was created and painted from BioRender.).

In summary, nanomaterials, given their excellent material properties and functionalization advantages, cannot directly promote only the process of osteogenic differentiation. They can also serve as efficient carriers to achieve targeted delivery of drugs, bioactive factors, and noncoding RNAs (e.g. lncRNAs, siRNAs, and microRNAs) to accurately modulate key signaling pathways related to bone regeneration, including the Wnt/β-catenin signaling pathway and the BMP-Smad signaling pathway (Figure 3 and Table 1).

**Table 1.** Nanomaterials mediate osteogenesis-related signaling pathways in promoting osteogenic differentiation.

Materials	Safe dosage	Toxicity verification	Encapsulators	Model	Targeting pathway	index	Effects	Ref. No.
FBS EXO	0.1 μg/mL	Good	Icariin	MC3T3-E1 cell line	—	BMP−2↑、RUNX2↑、OPN↑	Promote OB differentiation	(Dong et al. 2021)
CaO-rich BGN	1000 μg/mL	Non-toxic	—	Rat primary BMSCs	—	Runx2↑、Osx↑、ALP↑	Promote OB differentiation	(Meng et al. 2024)
HCCP-modified PAD	≤80 μg/mL	Non-toxic	Cur	MC3T3-E1 cell line	—	Runx2↑、OSX↑、ALP↑、COL1A1↑	Promote OB differentiation	(Yang et al. 2023)
PEI-coated MSN	≤100 μg/mL	Non-toxic	SOST siRNA and osteostatin	Mouse embryonic fibroblast (MEF) /OVX mice	Wnt/β-catenin	ALP↑、Runx2↑	Promote OB differentiation	(Mora-Raimundo et al. 2019)
Chitosan-conjugated AuNPs	≤10 ppm	Non-toxic	—	hADMSCs	Wnt/β-catenin	ALP↑、BSP↑、OCN↑	Promote OB differentiation	(Choi et al. 2015)
BMP−2 peptide functionalized MSN	50 μg/mL Or 100 μg/mL	Good	DEX	Rat primary BMSCs	BMP-Smad	Runx2↑、OCN↑	Promote OB differentiation	(Zhou et al. 2015)
IONP	≤400 μg/mL	Slightly toxic	—	Human primary BMSCs	BMP-Smad	INZEB2↑、ZEB2↓、Runx2↑、OSX↑、ALP↑、OPN↑、COL1A1↑	Promote OB differentiation	(Wang et al. 2017)

### 3.3 Precise inhibition of osteoclastic activity: a strategy for nanomaterial-mediated regulation of the NF-κB signaling pathway

During OC differentiation, the binding of RANK receptors and ligands on its surface regulates the main pathway of its differentiation (Sigl et al. 2016). RANKL ligands can trigger intracellular signaling pathways, such as the NF-kB, MAPK, and PI3K, in combination with receptors on the surface of OPC membranes, which in turn leads to the maturation, proliferation, and differentiation of OCs (Asagiri and Takayanagi 2007; Negishi-Koga and Takayanagi 2009). Therefore, specifically blocking the activation of its related pathways is the key to inhibiting OC generation.

The chemokine receptor (CXC chemokine receptor 4, CXCR4) is a receptor for stromal cell-derived factor 1 (SDF-1) and a key protein present in bone marrow (Dar et al. 2005). A selective and massive distribution of CXCR4 exosomes in the bone marrow can be observed when CXCR4 is encapsulated in NIH-3T3 cell-derived exosomes (Hu et al. 2021). After the overexpression of CXCR4 and receptor activators capable of scavenging RANKL ligands on the membranes of mouse BMSCs, Cui et al. extracted and encapsulated the cellular membranes of the BMSCs on the surface of pH-sensitive CS-based nanogels containing synthetic PTH1-34 and successfully constructed a biomimetic nanogel (PNG@m&C) (Cui et al. 2024). PNG@m&C exhibited significant targeting ability, which effectively blocked the RANKL-induced activation of the NF-κB pathway and inhibited the expression of the OC differentiation-related transcription factors c-fos and Nfatc1, as well as the specific genes Trap and Ctsk, thereby preventing the differentiation of BMM into OC. In addition, PNG@m&C achieved PTH1-34 release in the OC-mediated acidic environment, promoting osteogenic differentiation and restoring bone metabolic homeostasis, which significantly improved bone microarchitecture and reversed bone loss in mice.

Mature OCs cause acidification of the local microenvironment by attaching to the bone surface and secreting large amounts of hydrogen ions, which exacerbates the bone resorption effect of OCs and triggers degradation and erosion of the bone matrix. Therefore, improving the acidic environment around OCs is essential to alleviate the abnormal bone resorption process. Lin et al. developed a tetracycline-functionalized nanoliposome and encapsulated sodium bicarbonate (NaHCO_3_) within it to confer weak basicity, thus constructing functionalized nanocomplexes (NaHCO_3_-TNLs) (Lin et al. 2020). NaHCO_3_-TNLs reached the bone surface under the targeting effect of tetracycline, remodeling the local microenvironment through chemical modulation, initiating a biological cascade reaction, and reducing the bone resorption effect of OC. In this process, NaHCO_3_-TNLs first released NaHCO_3_ to neutralize the surrounding acidic microenvironment and inhibit the bone resorption function of OC. Disruption of the local acidic microenvironment induced damage and apoptosis of OC structures (Gerry and Leake 2014; Teti et al. 1989). Subsequently, apoptotic OCs released substantial amounts of RANK-containing EVs, which entered the extracellular microenvironment to bind to free RANKL, deplete the source of RANKL ligands, and prevent the activation of the NF-κB signaling pathway, thereby inhibiting OC proliferation, differentiation, and maturation (Veerman et al. 2019; Poon et al. 2019; Dirckx et al. 2019).

Moreover, autophagy plays an important role in maintaining bone homeostasis, and various autophagy-related genes are involved in regulating the process of OC differentiation (DeSelm et al. 2011). Jamie et al. treated OPCs with silica-activated nanomaterials (SINPs), which rapidly internalized OPCs within 24 h and significantly inhibited NF-κB and MAPK signaling pathway-induced OC differentiation (Arnst et al. 2023). In terms of the molecular mechanism, SINPs reduced the phosphorylation of the RANKL-induced kinases IκBα, IKKα, IKKβ, and JNK after being internalized, inhibited the expression of key downstream transcription factors, and inhibited early OC differentiation. Moreover, SiNPs stimulated the expression of the autophagy-related genes p62 and LC3β, which are dependent on the ERK1/2 signaling pathway. Interestingly, while stimulating autophagosome formation, SiNPs inhibited the autophagic flux required for RANKL-induced osteoclastic differentiation, thereby suppressing OC generation.

Gold nanoparticles (GNPs) have been demonstrated to inhibit the fusion and differentiation of monocyte macrophages into OCs, while relatively few studies have been conducted on the application of silver nanomaterials (SNPs) in bone regeneration (Sul et al. 2010). To compare the effects of GNPs and SNPs in inhibiting OC differentiation, Lee et al. treated BMM induced by RANKL and M-CSF with GNPs and SNPs, respectively (Lee et al. 2021). The results showed that SNPs were more effective at inhibiting the expression of the key transcription factors involved in OC differentiation, c-Fos and NFATc1, compared with GNPs. In addition, SNPs were more effective at inhibiting the phosphorylation of IkBa, ERK, JNK, and P38 in the RANKL-induced NF-κB and MAPK signaling pathways, resulting in consistently lower levels associated with OC differentiation in the SNP group than in the GNP group. Notably, SNPs did not exhibit cytotoxic effects while inhibiting OC differentiation, suggesting that they are an effective means to inhibit OC activity and function as safe and effective nanomaterials.

**Figure 4.**
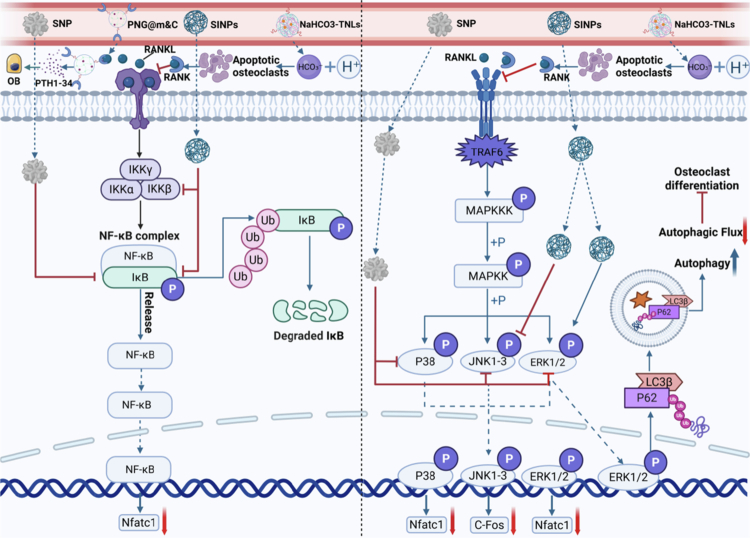
Application of nanomaterials for inhibiting osteoclast signaling pathways**.** PNG@m&C surfaces overexpress CXCR4 and RANK, enabling targeted suppression of the NF-κB signaling pathway. NaHCO₃-TNLs induce osteoclast apoptosis by neutralizing the acidic microenvironment and releasing RANK to further inhibit signaling. SINP and SNP suppress osteoclast differentiation pathways by reducing kinase phosphorylation in the NF-κB and MAPK/JNK signaling pathways. Additionally, SiNP inhibits the autophagic flux required for osteoclast differentiation, thereby impeding osteoclast formation. (This image was created and painted from BioRender.).

In summary, these nanomaterials have significant advantages in regulating OC activity and inhibiting its differentiation. These compounds not only have good biocompatibility and avoid toxic effects on other cells or organs but also effectively inhibit the differentiation and bone resorption effects of abnormal OC by targeting the RANKL-induced NF-κB and MAPK signaling pathways and blocking the activation of these pathways. These properties make nanomaterials unlimited potential in the clinical treatment of bone diseases (Figure 4 and Table 2).

**Table 2.** Nanomaterials inhibit osteoclast differentiation and activity by regulating osteoclast-related signaling pathways.

Materials	Safe dosage	Toxicity verification	Encapsulators	Model	Targeting pathway	Index	Effects	Ref. No.
BMSC cell vesicle-modified PNGs containing RANK and CXCR4	≤500 μg/mL	Good	PTH1-34	Mice primary BMMs/OVX mice	NF-κB	RANKL↓、c-Fos↓、Nfatc1↓、Trap↓、Ctsk↓、Runx2↑、OSX↑、ALP↑、COL1A1↑	Promote osteogenesis and inhibit osteoclastic activity	(Cui et al. 2024)
Tetracycline-modified DSPE-PEG	≤1.63 × 10^9^ particles/mL	Good	NaHCO_3_	Mice primary BMMs/OVX mice	NF-κB	RANK↑、β-CTX↓、TRAP↓	Promotes osteoclast apoptosis and inhibits osteoclast activity	(Lin et al. 2020)
SINP	—	Good	—	RAW264.7 cell line	NF-κB、MAPK、ERK1/2	Phosphorylation of IκBα,IKKα,IKKβ, and JNK↓、 Nfatc1↓、RANK↓、TRAP↓、p62↑、LC3β↑、 Autophagic flux↓	Inhibits osteoclastic differentiation	(Arnst et al. 2023)
SNP	≤0.05 nM	Good	—	BMMs	NF-κB、MAPK	c-Fos↓、NFATc1↓、Phosphorylation of IkBa, ERK, JNK, and P38↓	Inhibits osteoclastic differentiation	(Lee et al. 2021)

### 3.4 Immunometabolic reprogramming: nanomaterials target macrophage polarization to balance bone immune homeostasis

Osteoimmunology refers to the interactions and cross-regulatory mechanisms that exist between the skeletal system and the immune system (Srivastava and Sapra 2022), and the dynamic homeostasis of immune cells plays a critical role in bone homeostasis (Sapra et al. 2021). Immune cells can secrete various cytokines (e.g. RANKL, IL-6, and TNF-*α*) that significantly enhance bone resorption in OC. The inflammatory microenvironment triggered by bone diseases affects the functional status of immune cells, inducing them to perform adaptive regulatory and protective functions. However, the dysfunction of immune cells can directly or indirectly disrupt the dynamic balance between bone formation and bone resorption, leading to abnormal bone metabolism and related diseases. Strategies for achieving bone regeneration and repair based on bone immunoregulatory mechanisms are of great scientific significance and application value. Teriparatide is a bone anabolic drug used for the treatment of OP. One study revealed that intermittent teriparatide administration increased the number of regulatory T cells (Tregs) in mice to 2−3 times the original number (Yu et al. 2018). However, prolonged oral or injected administration of thyroid hormones such as teriparatide may be associated with many adverse effects, including headache, nausea, and diarrhea (Rizzoli and Reginster 2011). The stability, controlled release ability, and targeted delivery function of nanomaterials can largely prevent the above adverse effects, thus realizing a modulating effect on bone immunity (Dayanandan et al. 2023). In this section, the research strategy of the nanotechnology-mediated immune microenvironment for regulating bone metabolism is discussed in depth to provide prospective directions for the treatment of bone-related diseases, such as OP.

Macrophages are an important component of the immune system and one of the key pathways involved in the regulation of bone regeneration. mø can differentiate into two types of macrophages with specific differentiation functions: M1 and M2 types. M1-type macrophages are responsible for scavenging intracellular pathogens and secreting proinflammatory cytokines, such as TNF-*α*, IL-1, and IL-1β, which activate OC-related pathways and promote OC differentiation (Yamaguchi et al. 2016). In contrast, the anti-inflammatory effect of M2-type macrophages occurs mainly through the secretion of cytokines such as IL-4, IL-10, and CD206, which promote the differentiation and regeneration of OB (Zhuang et al. 2019). Lithium-doped calcium silicate cement has an extremely strong immunomodulatory effect, inducing a decrease in proinflammatory cytokines and polarization toward an anti-inflammatory M2 phenotype, and this dual effect promotes osteogenic differentiation (Lin et al. 2025). Thus, the induction of the polarization of M1 macrophages to M2 macrophages has become a potential target for the treatment of OP.

AuNPs have shown great potential in the treatment of inflammatory bone diseases (Higby 1982). Bai et al. found that AuNPs exerted favorable osteogenic effects on mice with lipopolysaccharide (LPS)-induced inflammatory bone erosion (Bai et al. 2021). Compared with that in the LPS group, the percentage of M2 macrophages in the bone tissue increased as much as threefold in the AuNP group, and the BMD of the mice increased by 14%. These results suggested that AuNPs can induce the macrophage phenotype to undergo M1-to-M2 polarization, thereby exerting an anti-inflammatory effect and improving the bone microstructure in mice in vivo. In recent years, HAP has been shown to regulate bone metabolism by regulating the bone immune microenvironment, but the inherent brittleness of its ceramic material limits the clinical application of HAP to some extent. To overcome this limitation, Chen et al. (Yang et al. 2019) successfully prepared nHAP by nanosizing HAP bioceramics using photolithography combined with a hydrothermal method. They found that nHAP could flexibly regulate immune cell responses and promote macrophage polarization from the M1 to M2 by nHAP treatment of macrophages (RAW 264.7 cells). After culturing human BMSCs (hBMSCs) using the supernatant obtained by centrifugation of the above macrophages, researchers cultured hBMSCs using the supernatant of M2 macrophages and found that the M2 macrophage microenvironment significantly upregulated the expression of osteogenesis-related genes (e.g. BMP-2, COL1, and Runx2) and facilitated the differentiation of hBMSCs into OBs. These results indicate that the M2 macrophage-mediated anti-inflammatory microenvironment can directly regulate OB differentiation.

In addition, the physical properties of nanomaterials (e.g. pore size, structure, and dimensions) significantly influence on the regulation of the bone immune microenvironment. In particular, the pore size and structure of nanomaterials affect the shape, adhesion, and spreading of macrophages, thereby modulating their inflammatory response of macrophages. For example, porous anodic aluminum oxide nanomaterials with different pore sizes altered the morphology of macrophages and activated their autophagic processes. Nanomaterials with larger pore size resulted in a decrease in the expression of M1 proinflammatory phenotypic markers and an increase in the expression of M2 anti-inflammatory phenotypic markers. This shift in macrophage phenotype further promoted osteogenesis-associated cytokines release of BMP and Wnt10b, activating the Wnt and BMP signaling pathways and promoting OB differentiation (Chen et al. 2017). Shen et al. evaluated the effect of nanomaterial size on the immunomodulation of osteogenic differentiation and found that large-scale TNT (TNT110) possessed significant antioxidant capacity (Shen et al. 2019). They verified by molecular and cellular experiments that, compared with 30 nm nanotubes, TNT110 activated the integrin/focal adhesion kinase (FAK)-mediated MAPK and NF-κB signaling pathways more significantly, thereby enhancing early inflammatory responses, as evidenced by the upregulated expression of SDF-1, IL-8, and chemokine (C–C motif) ligand 2. These early inflammatory events do not persist but rather recruit more MSCs, promote the conversion of M1 macrophages into M2 macrophages, eliminate early M1-mediated inflammatory phenotypic markers, increase intracellular oxidative stress levels, and increase osteogenic potential. The roughness of the surface of nanomaterials and their hydrophilic or hydrophobic properties also affect macrophage phenotypic polarization. High surface roughness has been shown to promote macrophage polarization toward the M2 phenotype, thus exerting an inhibitory effect on OC activity (Barth et al. 2013). Similarly, nanomaterials with surface hydrophilicity induce macrophage adhesion and M2-type polarization, whereas hydrophobic surface modifications altered the adhesion of monocytes and induced their polarization toward the M1 type (Li et al. 2020; Hotchkiss et al. 2016). These findings emphasize the important role of the physical properties of nanomaterials in bone metabolism by modulating the bone immune microenvironment.

In the context of immune modulation of bone metabolism, macrophages are usually the main target cells for nanomaterial delivery. Through the functional molecules they carry, they can modulate the inflammatory microenvironment and promote the polarization of proinflammatory M1-type macrophages to anti-inflammatory M2-type macrophages. IL-4, an anti-inflammatory cytokine, can modulate the polarization of M2 macrophages in vivo, alleviate tissue inflammation, and reduce the apoptosis of OBs (Wu et al. 2016). He et al. prepared high-stiffness transglutaminase nano-crosslinked gels (TG-gels) and encapsulated IL-4 in TG-gels, which significantly induced the movement of macrophages toward M1 macrophages in vitro, as well as macrophage polarization toward an M2 anti-inflammatory phenotype, thereby promoting the osteogenic differentiation of BMSC (He et al. 2019). In vivo experiments also demonstrated that TG gels preloaded with IL-4 enhanced bone regeneration in periodontal defect model mice. Niu et al. developed novel hollow cerium oxide NPs as carriers and coupled them with a tissue protease inhibitor (CA-074Me) to construct a hCeO2@CA-074Me NP composite system (Niu et al. 2025). In the *Porphyromonas gingivalis* LPS-induced macrophage inflammation model, the hCeO2@CA-074Me NPs effectively reduced the generation of reactive oxygen species (ROS) and decreased the level of the intracellular inflammatory microenvironment, which in turn facilitated the polarization of macrophages from M1 to M2. In terms of the molecular mechanism, the complex exhibited significant anti-inflammatory effects, mainly by inhibiting the CTSB-NLRP3 signaling pathway, thus reducing the release of the downstream inflammatory factors IL-18 and IL-1β and decreasing the expression of NLRP3 inflammatory vesicles. This anti-inflammatory microenvironment was favorable for OB maturation and differentiation. The application of nanocarriers can reduce the toxic effects of drugs on nontarget organs and significantly enhance the targeting and biosafety of drugs in vivo. BCL, a bioactive flavonoid extracted from the traditional Chinese herb *Scutellaria baicalensis*, has been shown to effectively inhibit the maturation process of OC and regulate bone metabolic homeostasis (Zhao et al. 2016; Saul et al. 2019). BCL has also shown significant anti-inflammatory effects, inducing macrophage polarization toward M2, thereby improving the inflammatory microenvironment (Teng et al. 2020). Wu et al. successfully developed a dual-targeted magnetic composite nanomaterial (BCL@MMSNPs-SS-CD-NW) loaded with BCL (Zhou et al. 2021). The material significantly enhances the delivery efficiency of BCL through the Fe_3_O_4_ magnetic targeting function, as well as the active targeting of NW peptides. Once BCL@MMSNPs-SS-CD-NW entered the mice, it efficiently enriched the bone defect site under the guidance of the applied magnetic field. The NW peptide further acted to induce the uptake of BCL by macrophages and promoted macrophage polarization toward the M2 phenotype, which ultimately inhibited the bone resorption function of OC at the site of bone injury and promoted bone regeneration.

The combined use of nanomaterials and other bioscaffolds is currently a central tool in clinical bone immunomodulation. Polyetheretherketone (PEEK) is a stable polymer that can be used as an implant material for bone defects given its good mechanical properties (Gao et al. 2023). However, because of the bioinertness of PEEK, prolonged hosting in tissues may lead to phytolysis and increase the risk of bone lesions (Gu et al. 2020). Therefore, improving the bioactivity of PEEK to enhance osseointegration ability is an important means to solve the above challenges. Liu et al. prepared CS-coated Mg–BGN nanocomplexes by codissolving BGNs with magnesium (Mg) metal powders in a CS solution (Liu et al. 2023). The complex was loaded into PEEK biomaterials by stirring, and multifunctionalized PEEK implants were successfully constructed. In vivo studies revealed that the functionalized PEEK implant had good bioactivity and osteointegration ability. In vitro cellular experiments further demonstrated that the scaffold achieved sustained release of Mg ions, which enhanced the osteogenic properties and bone mineralization at the site of intramedullary bone defects in rats. This effect was attributed to the fact that Mg ions effectively induced macrophage polarization toward an anti-inflammatory M2 phenotype by modulating the bone immune microenvironment while increasing the expression of the osteogenic-specific marker ALP and promoting the differentiation of BMSCs toward osteogenesis. In summary, PEEK multifunctional implant materials exhibit excellent bioactivity, biocompatibility, and osseointegration properties, making them an efficient solution for bone defect repair.

In addition to mediating the macrophage biological pathway, immunotherapy targeting T lymphocytes is a promising strategy. In estrogen deficiency-induced OP, overactivation of T cells is a key factor mediating inflammation and promoting osteoclastic resorption (Weitzmann and Pacifici 2006; Zhang et al. 2020). Specifically, the skeletal inflammatory microenvironment generated by estrogen deficiency induces T cell activation and disrupts the Treg/helper T cell 17 (Th17) balance; this leads to a decrease in Treg cells, which have the function of inhibiting osteoblastic differentiation; an increase in Th17 cells; and the secretion of IL-17, which induces the maturation and differentiation of OCs and exacerbates bone loss (Weitzmann and Pacifici 2006; Geng et al. 2017). Therefore, elimination of overactivated T cells under estrogen-deficient conditions is a key measure to remodel the bone immune microenvironment. MCP-1 is a key mediator of inflammation that induces T cells to migrate to the inflammation site (Yadav et al. 2010). Fas, a transmembrane protein and a member of the tumor necrosis factor receptor superfamily, is expressed mostly in activated T cells and can interact with Fas ligand (FasL) to initiate an apoptotic signaling pathway to induce T cell apoptosis (Strasser et al. 2009). To avoid the effects of Th17 cell activation, Yang et al. designed a T cell-depleted MSN, which, by encapsulating MCP-1 in the MSN and coupling it with FasL, accomplished T cell recruitment and initiated apoptosis in OVX mice, thereby eliminating activated T cells, establishing a new approach to immune homeostasis, improving BMD, BV/TV, Tb.N, and Tb.Sp, and alleviating bone loss in OVX mice (Yang et al. 2021). This study provides a promising therapeutic strategy for other immune diseases caused by overactivated T cells. In addition to its role in pathological sexual bone resorption, T cell activation can modulate osteogenesis (Könnecke et al. 2014). Crose et al. showed that soluble factors produced upon the activation of specific T cell subsets can influence the process of OB maturation, which is related to IL-17 (Croes et al. 2016). Xia et al. utilized mesoporous silica nanomaterials capable of mediating adaptive immune responses to regulate bone metabolism and achieve bone regeneration in cranial defect mice (Xia et al. 2023). The intrinsic mechanism is that silica ions released by MSN reduced the expression of regulatory factor X-1 in CD4 + T lymphocytes, leading to the overexpression of IL-17 by promoting histone H3 acetylation and reducing DNA methylation and H3K9 trimethylation. Subsequently, IL-17 enhanced the expression of the osteogenic transcription factor Runx2, the specific markers ALP and OCN, and the angiogenic gene expression. Thus, the mechanisms by which the adaptive immune system and its associated cytokines regulate bone regeneration are extremely complex. The specific molecular mechanisms by which T cells participate in OB maturation through direct or indirect pathways have not been fully elucidated and still require in-depth research and validation. Future research directions may focus on their active role in regulating bone metabolism through different lymphocyte subpopulations and their secreted cytokine networks.

**Figure 5.**
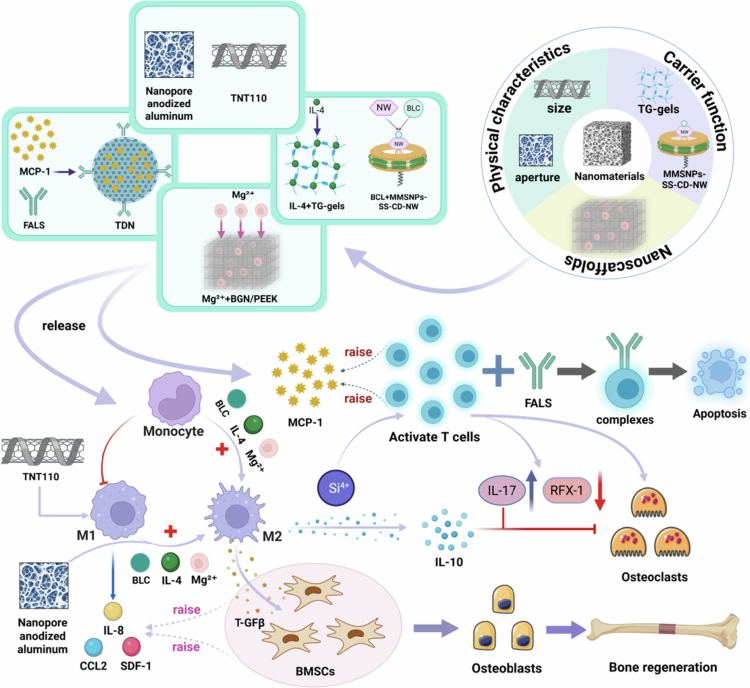
Nanomaterials mediate bone metabolism through regulation of the bone immune microenvironment. This section elucidates how nanomaterials serve as multifunctional platforms. Through multiple pathways – including their physical properties (pore size/dimensions), delivery of bioactive factors (encapsulation of IL-4 and BCL), scaffold-mediated release of active ions (Mg²⁺ release), and T-cell immunomodulation – they induce macrophage polarisation towards the M2 phenotype. This process inhibits bone resorption and accelerates bone formation. (This image was created and painted from BioRender.).

In summary, the immune system and the skeletal system are inextricably linked. In particular, macrophages play an important role in affecting bone regeneration. Nanomaterials can influence the immune-regulatory response through their own properties, carrier delivery, and scaffolding strategies, thus realizing the regulation of bone metabolism, which provides a new research idea and scientific basis for the clinical treatment of bone diseases (Figure 5 and Table 3).

**Table 3.** Nanomaterials mediate bone immune microenvironment to regulate bone metabolism.

Materials	Safe dosage	Toxicity verification	Encapsulators	Model	Targeting pathway	Index	Effects	Ref. No.
AuNPs	≤3 μg/mL	Non-toxic	—	RAW264.7 cell line/LPS-induced femur erosion mice	—	M2 macrophages↑、CD80↓、CD206↑、COL1A1↑	Inhibition of persistent inflammation, BMD increased by 14%	(Bai et al. 2021)
Lithography combined with hydrothermal method to nanoscale HAP bioceramics	—	—	—	RAW264.7 cell line/hBMSCs	—	M2 macrophages↑、BMP2↑、Runx2↑、COL1A1↑	Improve the inflammatory microenvironment and promote osteogenic differentiation	(Yang et al. 2019)
Large size TNT_110_	—	Low toxicity	—	RAW264.7 cell line/Mice primary MSCs	MAPK、NF-κB	Early stages: SDF−1↑、IL−8↑、CCL2↑ Late stage: M2 macrophages↑、IL−4↑、IL−10↑、IL−13↑、TGFβ1↑、ALP↑、COL IA1↑、OPN↑、OCN↑	Promotes osteogenic differentiation	(Shen et al. 2019)
Transglutaminase-modified TG-gels	—	—	IL−4	RAW264.7 cell line/Rat primary MSCs	—	M2macrophages↑、 Arg↑、 CD206↑	Improve the inflammatory microenvironment and promote osteogenic differentiation	(He et al. 2019)
NW peptide-modified MMSNPs	≤5.3 mg/mL	Good	BCL	Mice Primary MSCs/BMMs/Femur fracture mice	NF-κB	M2macrophages↑、TNF-α↓、IL-1β↓、Runx2↑、COL1A1↑、OCN↑	Produces an anti-inflammatory effect and promotes osteogenic differentiation	(Zhou et al. 2021)
chitosan-coated PEEK/BGN	≤0.1 mg/mL	Good	Mg^2+^	Raw 264.7 cell line/rBMSCs/Femoral defects rat	—	M2macrophages↑、CD206↑、ALP↑、Runx2↑、BMP2↑、OCN↑、OPN↑	Improve the bone immune microenvironment and enhance osteogenic properties	(Liu et al. 2023)
FasL-modified MSN	—	—	MCP	Activated spleen T cells/Mice primary MSCs/OVX mice	—	Runx2↑、ALP↑、IFN-γ↓、IL−17↓、TNF-α↓	Activates T cell apoptosis, enhances anti-inflammatory effect, and improves osteogenic differentiation ability	(Yang et al. 2021)
MSN	10–150 µg/mL	Good	—	CD4 T lymphocytes/Mice primary BMSCs/Cranial defect mice	—	RFX−1↓、DNA methylation↓、H3K9 trimethylation↓、IL−17↑、IL−6↑、Runx2↑、ALP↑、OCN↑	Regulates CD4 T lymphocyte adaptive immune response and promotes osteogenic differentiation	(Xia et al. 2023)

### 3.5 Vascular-osteogenic coupling effects: nanocarrier-mediated targeted formation of H-vessels

The bone vasculature plays an important role in bone homeostasis and repair, which not only provides bone tissue with the required nutrients, oxygen, growth factors, and hormones but also participates in the regulation of osteogenic signaling molecules to perform osteogenic functions (Peng et al. 2020; Street et al. 2002). The bone vasculature comprises two main types: H and L types (Sivan et al. 2019). Among them, H-type bone vasculature is a unique microvascular component with high expression of platelet and EC adhesion molecule 1 and endomucin, which are essential in the coupling of angiogenesis and osteogenesis (Zhang et al. 2020; Kusumbe et al. 2014). In an aged mouse model, reduced local blood flow led to a significant reduction in H-vessels and endothelial sprout structures near the growth plate, accompanied by a significant decrease in the number of osteoprogenitor cells (OSCs) expressing Osx. In contrast, the number of OCs did not significantly change. By reactivating the Notch signaling pathway in ECs, H-vessel formation can be restored, and the recruitment of OSCs can be promoted, thereby reversing the degradation of the bone microenvironment in aging mice (Ramasamy et al. 2016). These findings revealed a close coupling between angiogenesis and osteogenesis and emphasized the critical role of vascular–osteogenic coupling in bone regeneration. Deferoxamine (DFO), an iron chelator, can stabilize and activate the hypoxia-inducible factor-1α (HIF-1α) signaling pathway by inhibiting prolyl hydroxylation of HIF-1α, which in turn promotes downstream transactivation of vascular endothelial growth factor (VEGF) and ultimately induces H-type angiogenesis (Park et al. 2022; Ngo and Harley 2020). However, the clinical application of DFO is restricted by its short half-life and its ability to be easily cleared by the body; thus, there is an urgent need for the development of an efficient nanosystem for its delivery (Liu et al. 2021). Li et al. succeeded in constructing a zeolitic imidazolium ester skeleton-8-based three-dimensional nanoframework (DFO@ZIF-8 NPs), in which Zn^2+^ was linked to 2-methylimidazole, which formed stable nanostructures through coordination. DFO@ZIF-8 NPs efficiently loaded and delivered DFO to target cells through their excellent biocompatibility, high porosity, and tunable pore sizes, exhibiting the remarkable osteogenic properties of this nanosystem. Specifically, DFO@ZIF-8 NPs first activated the PI3K-AKT signaling pathway by releasing Zn²⁺, which upregulated the expression of matrix metalloproteinase (MMP)-2 and MMP-9, leading to the degradation of the vascular basement membrane, thus initiating the initial angiogenic phase of angiogenesis (Li et al. 2023). Subsequently, DFO is released to activate the HIF-1α signaling pathway to increase VEGF expression and induce vascular ECs to differentiate into more H-type vessels. Meanwhile, the release of DFO can activate the MAPK signaling pathway, upregulate the expression of OCN and BMP-2, and ultimately promote osteogenic differentiation and bone matrix accumulation. The synergistic effect of Zn²⁺ with DFO significantly enhances angiogenesis–osteogenesis coupling, providing a highly efficient and translationally promising strategy for the treatment of skeletal diseases.

**Figure 6.**
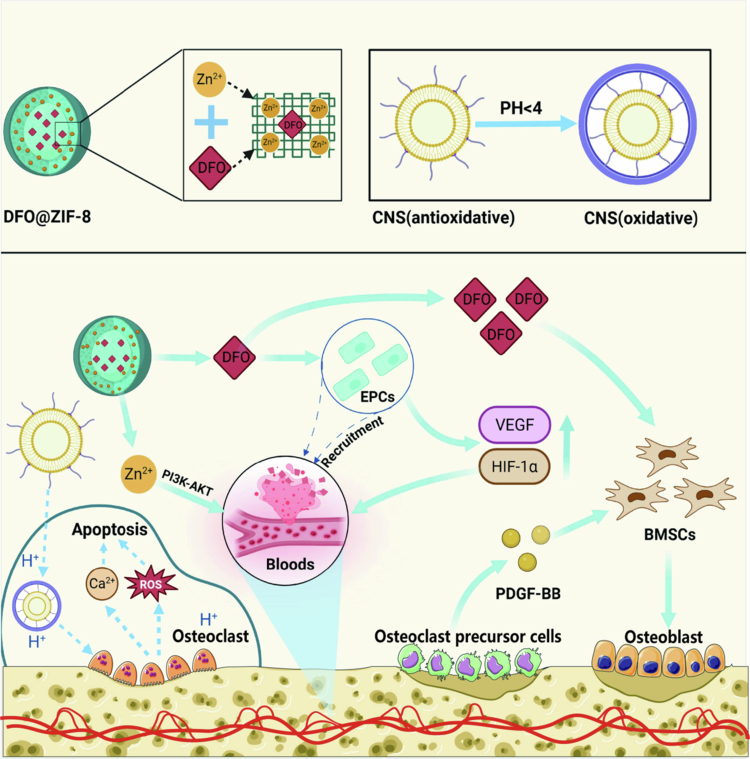
Nanomaterial-mediated vascular‒osteogenic coupling mechanism. (a) Schematic diagram of DFO@ZIF-8 and CNS nanosystems. (b) DFO@ZIF-8 NPs release Zn²⁺ to activate the PI3K-AKT pathway, upregulating MMPs to promote degradation of the vascular basement membrane and EC migration. The DFO payload of these proteins activates the HIF-1α and MAPK pathways, which induce H-type angiogenesis and osteogenic differentiation, respectively. CNS nanosystems target OCs and generate reactive oxygen species to inhibit their own activity. OPCs continuously release PDGF-BB, synergistically promoting angiogenesis and bone formation via the PI3K-Akt/FAK pathway. (This image was created and painted from BioRender.).

Recent studies have shown that OPCs can secrete platelet-derived growth factor-BB (PDGF-BB), which in turn enhances vascular–osteogenic coupling, a finding that provides a new therapeutic target for bone tissue regeneration strategies (Xie et al. 2014). On the basis of this mechanism, Zhang et al. designed a core–shell-structured NP, which was loaded with an siRNA targeting dendritic cell-specific transmembrane proteins, and modified this NP with alendronate to increase the recycling and bone-targeted delivery efficiency of the siRNA (Zhang et al. 2023). This system not only inhibited the generation of mature OCs and bone resorption but also preserved the ability of OPCs to secrete PDGF-BB, which induced H-type angiogenesis and the expression of the osteogenic marker OCN. This strategy successfully reversed bone loss in the ovariectomized mouse model. The bone-resorptive activity of OC is highly dependent on the secretion of acidic substances to achieve bone matrix dissolution in the mature stage, accompanied by significant oxidative stress properties. Mature OCs usually exhibit high levels of ROS, which enhance the bone-destroying function of OC through a positive feedback mechanism. Dou et al. developed a pH-targeted cerium nanosystem by utilizing the unique acidic environment of mature OCs (Dou et al. 2021). It can specifically migrate around mature OCs at relatively low pH values under the guidance of a carried pH-sensitive enzyme and induce excessive ROS generation within mature OCs through a shift in its enzymatic activity from being an antioxidant to being oxidative. These characteristics induce the accumulation of intracellular ROS bursts, trigger an abnormal increase in calcium oscillations, weaken the activity of mature OCs, and thus improve the surrounding acidic microenvironment. Notably, OPC was unaffected by pH changes, and the retained OPC continued to produce PDGF-BB and activate the PI3K-Akt signaling pathway and FAK, which synergistically promoted endothelial progenitor cell vasculogenesis and osteogenic differentiation of the BMSCs. In the OVX-induced OP mouse model, this strategy effectively achieves bidirectional synergistic reconstruction of vascular–osteogenesis through the inhibition of OC-mediated bone resorption and significantly alleviates pathological bone loss (Figure 6).

### 3.6 New dimension of lipid metabolism intervention: nanomaterials modulate the PPARγ pathway to reverse bone marrow steatosis

In recent years, the role of BMAT in the regulation of bone metabolism has received increasing attention, but its importance is still often underestimated. BMAs are one of the cell lineages differentiated from BMSCs, and together with OBs and chondrocytes, they constitute the multidirectional differentiation potential of BMSCs (Qiu et al. 2018). BMAs are capable of releasing various biologically active factors, including adipokines, free fatty acids, EVs, proinflammatory adipokines, and other regulatory proteins, such as RANKL and stem cell factor, thus exerting significant regulatory effects on bone metabolism and the hematopoietic microenvironment (Li et al. 2019). However, aberrant differentiation of BMSCs in patients with skeletal diseases leads to an imbalance between osteogenesis and adipogenesis, as evidenced by a decrease in the differentiation of OBs and an increase in the differentiation of adipocytes; this pathologically shifted differentiation can have a significant negative effect on bone matrix mineralization, exacerbating the progression and deterioration of the disease (Yao et al. 2023). Therefore, restoring the balance between OBs and BMAs is essential for bone tissue regeneration and remodeling.

PPARγ is a nuclear transcription factor that induces adipose progenitor cell-to-adipocyte differentiation together with the CCAAT enhancer-binding proteins α/β. MiR-188 is a microRNA that increases with aging, which significantly promotes adipogenesis and inhibits BMSC differentiation (Li et al. 2015). Designing an siRNA to target miR-188 is a therapeutic means to promote bone formation. Hu et al. fused liposome-encapsulated miR-188 siRNA with exosomes from NIH-3T3 cells highly expressing the CXCR4 gene, with the aim of allowing specific aggregation of hybridized NPs in bone marrow tissue (Hu et al. 2021). In vivo studies showed that hybridized NPs could reach the bone marrow precisely to release antagonist 188, which reduced the number of BMAs while increasing the number of bone trabeculae and alleviating age-induced bone loss. In vitro verification indicated that NP-treated BMSC significantly reduced the expression of PPARγ and fatty acid-binding protein 4 and inhibited bone marrow adipogenic differentiation. In contrast, the osteogenic differentiation-specific markers OSX and OCN were significantly upregulated, suggesting a significant improvement in the osteogenic differentiation of the BMSCs. Furthermore, oxidative stress plays an important role in promoting bone marrow adipogenesis. High levels of ROS can promote BMSC differentiation toward the adipocyte lineage by upregulating PPARγ expression, which in turn promotes BMSC differentiation toward the adipocyte lineage (Atashi et al. 2015). However, excessive accumulation of BMAT at bone defect sites enhances the bone resorption activity of OCs while inhibiting the repair and regeneration processes of bone (Justesen et al. 2001). Therefore, reducing ROS levels within BMSCs to the normal physiological range may be a potential strategy to promote bone regeneration. Ursodeoxycholic acid (UDCA) is a hydrophilic bile acid whose antioxidant capacity and anti-inflammatory activity can effectively promote osteogenic differentiation and inhibit lipogenic differentiation in BMSCs (Cha et al. 2016). Some studies have reported that long-term injection of UDCA may have certain side effects on body tissues and organs. To reduce the risk of the drug to other tissues, Arai et al. developed PUDCA NPs for bone regeneration, a nanosystem that is highly sensitive to H_2_O_2_, which can rapidly reach the site of bone defects and carry out sustained release of UDCA (Arai et al. 2020). It effectively reduces the ROS level, inhibits IL-1β expression, promotes the osteogenic differentiation of BMSCs, prevents the lipogenic differentiation of BMSCs, and enhances bone regeneration in the epiphysis and diaphysis of long bones in rats.

### 3.7 Nanotechnology-mediated neuromodulation for bone regeneration

Neuromodulation, an important driver of bone formation, spans the entire spectrum of skeletal physiologic activity from embryogenesis to remodeling and repair after maturation (Tomlinson et al. 2016). In general, the nerve fibers innervating bone are mainly sensory and sympathetic nerves, along with parasympathetic nerves. However, the pattern of inter-regulation and innervation of the skeleton and parasympathetic nerves is unclear and requires further study (Elefteriou 2018). These nerve fibers are derived from the differentiation of mesenchymal stem cells and are widely distributed in the microenvironment of bone tissues, such as cortical bone, trabecular bone, bone marrow, and periosteum, which together participate in the regulation of bone metabolism by OBs and OCs and maintain bone homeostasis (Mach et al. 2002). Sensory nerves in bone tissue not only interact with the central nervous system but also secrete tyrosine kinase receptor A to regulate bone development, remodeling, and repair processes (Brazill et al. 2019; Hassan et al. 2023). Sympathetic nerves can secrete the neurotransmitter norepinephrine, which interacts with β2-adrenergic receptors on the bone surface to negatively regulate bone formation (Takeda et al. 2002; Bonnet et al. 2005). Nerve fiber damage and deficiencies caused by bone defect diseases severely impede the regulatory effects of nerve fibers on bone metabolism, affecting bone quality and healing efficiency (Kingery et al. 2003). Previous studies in the field of bone regeneration have focused on strategies to promote the osteogenic differentiation of BMSC and enhance OB activity (Tang et al. 2018). Certain drugs are effective in promoting the recovery of the nervous system of bone tissue to enhance bone repair and regeneration(Huang et al. 2023). However, the possible side effects caused by long-term drug administration and the lack of targeted drugs have limited the effectiveness of the clinical application of these drugs. Therefore, by precisely delivering drugs to BMSCs and inducing their differentiation toward neuron-like cells, nanomedicine technology provides a potential idea to solve the above problems.

**Figure 7.**
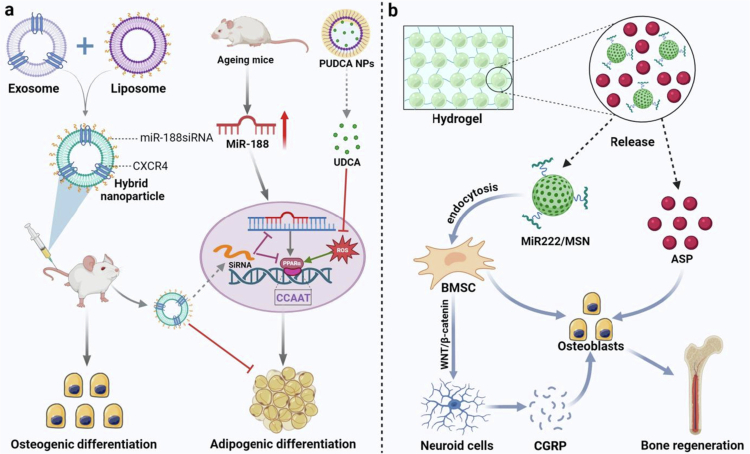
Nanomaterials reverse bone marrow adipogenesis and neurally modulate bone regeneration. (a) Hybrid miR-188 siRNA/CXCR4 nanoparticles targeting miR-188 siRNA effectively inhibited PPAR- and CCAAT-enhancer-binding protein α/β co-induced adipogenic differentiation. Oxidative stress-responsive PUDCA nanoparticles scavenge reactive oxygen species and downregulate IL-1β levels, thereby blocking the adipogenic differentiation. (b) The MiR-222/MSN/ASP hydrogel releases MiR-222 to activate the Wnt/β-catenin pathway, thereby promoting the differentiation of BMSCs into neurite-like cells. Concurrently, it synergistically enhances osteogenic differentiation potential through the secretion of CGRP and ASP. (This image was created and painted from BioRender.).

**Figure 8.**
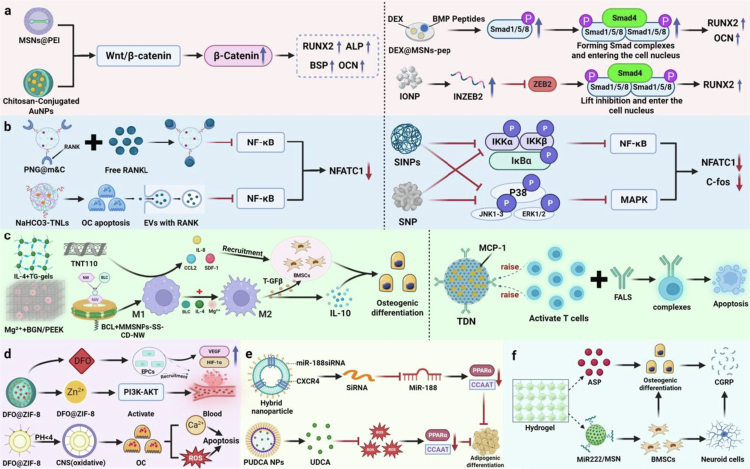
Integrated schematic illustration of the mechanisms and applications of nanomaterials in modulating the bone microenvironment. (a) Targeting the osteogenic microenvironment (b) Inhibiting the osteoclastic microenvironment (c) Modulating the immune microenvironment (d) Vascular‒osteogenic coupling mechanisms (e) Revering bone marrow adipogenesis (f) neurally regulated bone regeneration (this image was created and painted from BioRender.).

Lei et al. developed an injectable thermo-responsive MSN embedded in a core–shell structure of poly(ethylene glycol)-b-poly(lactic acid-hydroxyacetic acid copolymer)-b-poly(*N*-isopropylacrylamide) hydrogel in the form of a miR222/MSN/ASP hydrogel for the codelivery of miR222 and ASP, which was designed to achieve innervated bone regeneration function (Lei et al. 2019). MiR-222 is an endogenous small noncoding RNA that promotes neuronal growth and induces regenerative processes after nerve injury(Yu et al. 2012). In addition, miR-222 has shown potential applications in the field of innervated bone tissue engineering (Zhou et al. 2012). ASP is a nonsteroidal anti-inflammatory drug that can be used to reduce the inflammatory microenvironment and enhance the osteogenic potential of bone defect areas (Cao et al. 2015). The results showed that the miR222/MSN/ASP experimental group presented significantly upregulated the expression of neurogenic proteins and generated more bone tissue at the bone defect site compared with the MSN/ASP group. By contrast, ASP alone did not significantly promote osteogenesis in rat mandibular defects. Further mechanistic studies demonstrated that miR-222 induced BMSCs to differentiate into neural-like cells and secrete CGRP to promote the osteogenic differentiation of peripheral BMSCs through Wnt/β-catenin signaling. miR-222 could further increase the osteogenic potential of CGRP-stimulated BMSCs in the presence of ASP, thus accelerating this process of BMSC differentiation into OBs and creating a favorable environment for the realization of innervated bone regeneration. Nanomedicine, which has a precise delivery function, efficient drug-carrying capacity, and controllable slow-release kinetics, offers the possibility of nerve–bone regeneration and provides a new research direction for bone regeneration strategies (Li et al. 2024). However, few studies exist on innervated bone regeneration, which are still in the preliminary exploration stage (Wen et al. 2024). In addition, its clinical application faces many challenges, including its biosafety, in vivo targeting efficiency, and long-term biocompatibility of nanomaterials (Zhang et al. 2023). Therefore, the clinical translation of nanomedicine in the field of nerve–bone regeneration urgently needs to be promoted through in-depth mechanism research, technology optimization, and multicenter clinical validation to make a substantial leap from basic research to clinical practice (Figures 7, 8 and Table 4).

**Table 4.** The application of nanomaterials in multi-dimensional regulation of bone metabolism through blood vessel-fat-nerve.

Materials	Safe dosage	Toxicity verification	Encapsulators	Model	Targeting pathway	Index	Effects	Ref. No.
ZIF-8	≤60 μg/mL	Good	DFO/Zn^2+^	HUVECs/BMSCs/Cranial defect mice	PI3K-AKT/HIF-1α/MAPK	ALP↑、Runx2↑、Ocn↑、Bmp−2↑、MMP2↑、MMP9↑、VEGF↑	Vascular-osteogenic coupling promotes bone regeneration	(Li et al. 2023)
Surface PH-Sensitive enzyme-modified CNS	10–100 μg/ml	Low toxicity	—	RANKL-induced BMMs/OVX mice	PI3K-AKT	ROS↑、Ca^2+^↑、PDGF-BB↑、VEGF↑、Phosphorylation of PI3K, Akt, and FAK↑、P1NP↓、CTX↓	Induces OC cell apoptosis, enhances angiogenesis and osteogenesis	(Dou et al. 2021)
CXCR4-modified NIH-3T3 Exo/Liposomes	≤500 μg/mL	Good	miR-188siRNA	NIH-3T3 cell line/Age-related bone loss mice	PPARγ	PPARγ↓、FABP4↓、OSX↑、OCN↑	Inhibit adipocyte differentiation and promote osteoblast differentiation	(Hu et al. 2021)
PUDCA NP	107.52 µg/mL	Non-toxic	UDCA	hMSCs/Long bone defect rat	PPARγ	ROS↓、IL-1β↓、PPARγ ↓、RUNX2 ↑、ALP ↑	Inhibit adipocyte differentiation and promote osteoblast differentiation	(Arai et al. 2020)
PEG-PLGA-PNIAM-coated MSN	—	Non-toxic	miR222/ASP	hBMSCs/Mandibular defect rat	Wnt/β-catenin	MAP↑、NGF↑、NG2↑、NLK ↓、β-catenin↑、TNF-*α* ↓、 IFN-*γ* ↓、 CGRP↑	Promotes nerve differentiation and bone regeneration	(Lei et al. 2019)

## 4. Challenges

### 4.1 Standardized dilemmas in nanobio–interface interaction mechanisms and toxicity assessment

The primary key scientific issue facing the biomedical applications of nanotechnology is that its biosafety has not yet been fully elucidated. During the design and screening of nanomaterials, systematically assessing whether they can coexist harmoniously with the cells and tissues of living organisms is necessary to avoid triggering immune reactions or toxicity. Ideal nanomaterials should be able to degrade gradually after tissue regeneration, and the acute and chronic toxic effects of submicron fragments and residues (e.g. transition metals, such as Fe, Ni, and Co) generated during degradation need to be systematically evaluated to avoid potential adverse effects associated with long-term retention in the body. In addition, various factors, such as the size of the nanomaterials, the drug-carrying dose, and the exposure time, may affect the body’s metabolism, as well as biosafety. Once nanomaterials enter the human circulatory system, the surface charge of the materials may adsorb plasma proteins, triggering abnormal coagulation and erythrocyte lysis. Therefore, systematic elucidation of the pharmacological mechanisms of relevant nanomaterials on human health and the environment and the establishment of corresponding toxicity assessment criteria are necessary for the clinical translation of nanotechnology.

### 4.2 Dilemma of complex synthesis process and large-scale production of nanoproducts

The product transformation of nanomaterials faces multiple technical bottlenecks, mainly in the following aspects: First, at the level of the preparation process, the complex synthesis pathway leads to difficulty in accurately controlling the size distribution of NPs and their dispersion in solution, which directly affects the batch-to-batch consistency of the products (Zorrón et al. 2024). Second, significant challenges remain in balancing process reproducibility, cost-effectiveness, and the requirements of the current pharmaceutical manufacturing quality management standards during the scale-up process. Third, surface-modified functionalized nanomaterials may still trigger immune rejection and abnormal thrombosis, which constitute major barriers to the translational application of the products. Therefore, the implementation of the new generation of nanosystems for translation should improve the precision of product preparation as much as possible and follow the principle of ‘translatability’ to optimize the degradation kinetics, biocompatibility, and immune inertness of the materials while expanding the scale of production to reduce the potential risks of clinical applications.

### 4.3 Translational bottlenecks in nanomedicine: discrepancy between predictability and clinical validity of animal models

While the role of nanomaterials in regulating bone metabolism and regeneration has been demonstrated through in vitro cellular studies and small animal models, challenges occur in the translation from animal models to clinical trials. The reason is the complex physiological environment of clinical patients, which is considerably more complex than those of experimental animals and cellular models. Common experimental animals, such as rats and mice, have significantly smaller bone volumes than those of humans. Based on the data obtained from animal models and in vitro cellular experiments, including the permeation retention ability and tissue fusion ability of nanomaterials, which may differ from the results of clinical experiments, some limitations may lead to the overestimation of drug delivery ability. Therefore, further construction of experimental models resembling human physiological and pathological structures is necessary to evaluate the effectiveness of nanomaterials in the treatment of bone metabolic diseases.

## 5. Future outlook

### 5.1 Programmable nanoplatform construction based on multimodal synergistic therapy

Nanomaterial-based mediated bone microenvironment strategies provide a new perspective and direction for the treatment of bone metabolism, bone defects, bone metastasis, and other diseases. Currently, the most promising direction is combined therapy, in which multiple drug therapies are used in combination to construct a multifunctional nanodelivery system to restore bone microenvironmental homeostasis. This therapeutic strategy is not just a simple functional superposition, but rather a rationalization of nanomaterial modification to achieve the targeting of tissues and the orderly delivery of drugs to achieve the excellent effect of ‘one plus one is greater than two.’ For example, tetracycline-modified MSNs can target bone tissues by carrying DEX and bisphosphonates and exert synergistic therapeutic effects on the two to improve the therapeutic effect. In the future, a systematic modular platform will be built so that doctors can provide personalized treatment plans for thousands of patients based on each patient’s disease progression and needs.

### 5.2 Microenvironmental response-based controlled release system for nanocarrier delivery

The development of microenvironment-responsive nanocarriers is a core research direction in the field of precision drug delivery. By utilizing biomarkers specific to the diseased region (e.g. local pH gradients, temperature changes, or specific enzyme activities), nanocarriers can be designed to enable targeted delivery of pharmaceutical agents, thereby significantly reducing off-target effects and enhancing therapeutic efficiency. By responding to these cues, nanosystems can optimize the concentration and exposure time in the target region owing to their own kinetic release advantages, ultimately improving therapeutic efficacy while reducing systemic drug toxicity and side effects. Furthermore, the integration of computational modeling (e.g. molecular dynamic simulations) with machine learning algorithms provides a new paradigm for predicting and optimizing the release kinetics of nanocarriers. As a result, data-driven material design approaches allow intelligent delivery systems tailored to specific tissue microenvironments, thus significantly increasing the efficiency and therapeutic precision of nanomedicine interventions.

### 5.3 Nano-smart sensing platform based on biomarker regulation

Multidimensional regulatory strategies for the bone microenvironment will increasingly rely on advanced imaging techniques and computational modeling to elucidate the interactions between nanomaterials and cells. The above techniques can be used to elucidate the pathways by which nanomaterials in regulating immune response, lipogenic differentiation, microvessel formation, and nerve regeneration. In addition, nanomaterials, which have high porosity and specific surface area, can be ideal materials for the preparation of highly sensitive biosensors. Specifically, nanomaterials are selected as the substrate of electrochemical sensors by loading materials with conductivity and corresponding catalytic enzymes to obtain a high-precision electrochemical sensor. This smart sensor can accurately detect the concentration of the target area, as well as other indicators, and provide electrical signal feedback to external devices. This strategy can aid in the prevention and supervision of OP, osteoarthritis, and bone tumor diseases.

## 6. Summary

This review comprehensively summarizes the multidimensional regulatory roles of different types of nanomaterials in the bone microenvironment, including the induction of osteogenic differentiation, inhibition of osteoclastic activity, balancing of bone immunity, reversal of bone marrow steatosis, promotion of angiogenesis, and neural-osteogenesis. Among them, some nanomaterials can alter cell adhesion, migration, proliferation, and differentiation abilities on the basis of their own physical properties (e.g. pore size, structure, dimension, and hydrophilicity or hydrophobicity). In addition, nanomaterials can be used as carriers of drugs, genes, and bioactive factors to efficiently deliver piggyback substances to target cells or regions for precise regulation of the bone microenvironment. Nanomaterials can also mimic the natural structure of the bone tissue complex and the appropriate mechanical microenvironment by combining with other biomaterials to promote cell adhesion, proliferation, differentiation, and integration into surrounding tissues. Researchers have developed various functionalized nanodelivery systems and bioscaffolds, providing a theoretical basis for personalized therapies for bone-related diseases. Nanomaterials still face many challenges in clinical translation, such as material biocompatibility, potential cytotoxicity, and synthesis process complexity. Nevertheless, the unique advantages of nanomaterials in the treatment of bone diseases, including extremely high specific surface area, excellent drug-carrying capacity, and flexible surface functionalization, have been well established. Further studies are needed to pave the way for the use of nanotechnology in the clinical treatment of bone diseases in the future.

## Data Availability

Data sharing is not applicable to this article as no new data were created or analyzed in this study.
